# Editorial: Food derived bioactive metabolites: unlocking their potential health benefits and medical potential

**DOI:** 10.3389/fphar.2026.1772586

**Published:** 2026-01-28

**Authors:** Ruyu Yao, Massimo Lucarini, Alessandra Durazzo

**Affiliations:** 1 Yunnan International Joint Laboratory of Health Plant Resources Development and Department of Economic Plants and Biotechnology, Yunnan Key Laboratory for Wild Plant Resources, Kunming Institute of Botany, Chinese Academy of Sciences (CAS), Kunming, China; 2 CREA-Research Centre for Food and Nutrition, Rome, Italy

**Keywords:** bioactive metabolites, carotenoids, cellular signaling pathways, dietary supplements, disease modulation, ethnopharmacology, functional foods, nutraceuticals

## 1 Introduction

The “food and medicine continuum” is commonly seen in different geographic and biocultural backgrounds, as many food-derived metabolites also have health-promoting functions (Yao et al., 2023). Two decades ago, the study of edible medicines was reported as “an ethnopharmacology of food” by Etkin, (2006). In this context, the present Research Topic aims at investigating the intricate relationship between medicines and foods across various cultures and traditions. Components isolated from these food-medicines can serve preventative roles, support physiological functions, or act as targeted treatments due to their interaction with specific receptors. Given these overlapping domains, a comprehensive understanding of the scientific potential of food-derived metabolites is crucial. The present Research Topic, titled “Food-Derived Bioactive Metabolites: Unlocking their Potential Health Benefits and Medical Potential”, brings together several contributions. Articles were focused on the study of food-based metabolites and their bioactivities, highlighting their health-promoting potentials.

This Research Topic starts with a study on two vegetable species from Asia, namely, *Colocasia affinis* Schott and *Colocasia gigantea* Hook. f. by Alam et al. The authors isolated six bioactive phytochemicals from these two *Colocasia* species and elucidated their structures employing the NMR technique; tested their health functions, including antidiarrheal, analgesic, and anti-inflammatory, were then tested. Tian et al. identified 85 components from the fruit *Sinopodophyllum hexandrum* (Royle) Ying, which is mainly distributed in the Himalayan alpine region, and explored the *in vivo* transformation of their chemical components. Their anti-tumor activities were studied with network pharmacology and provided new insights into developing natural anti-tumor agents. Olas presented a mini review on an African species, marula [*Sclerocarya birrea* (A. Rich.) Hochst.], and its products, with a special emphasis on their chemical composition, biological activity, and health-promoting potential. Jakimiuk et al. reported the *ex vivo* biotransformation of lady’s mantle extracts via the human gut microbiota, with focus on the formation of phenolic metabolites and their impact on human normal and colon cancer cell lines. The genus *Elsholtzia* Willd. comprises fragrant food-medicinal species, and Inta et al. reported on the species found in Northern Thailand, using ethnobotany, chemical analysis, and anti-glycation activity, providing important clues for the development of food-medicinal uses of this taxon.

Several globally popular species are also studied in this Research Topic. The genus *Citrus* L. includes many of the common edible fruits, and their peels are a rich source of flavonoids; Xu et al. summarizes the types, bioactivities, and mechanisms of action of *Citrus* flavonoids, providing scientific evidence for their research and development. Du et al. reviewed the bioactivity and biomedical applications of pomegranate (*Punica granatum* L.) peel, which is rich in various bioactive metabolites such as polyphenols, tannins, and flavonoids, showing high medicinal and nutritional value. Liu et al. conducted a systematic review on the pharmacology and mechanism of action of *Monascus purpureus* Went, providing a reference for future study. From a perspective of sustainability, Marrone et al. discussed the important role of plant-based diets in human health, with an emphasis on chronic kidney disease prevention and treatment.

Moreover, bioactivities of specific compounds have also been studied. Luteolin is a flavonoid widely found in food plants; Lv et al. summarized the research advancements in improving the solubility and bioavailability of luteolin, and discussed its therapeutic effects in the treatment of pulmonary diseases. In the study by Enkhbat et al., grifolin and grifolic acid were isolated from the edible mushroom *Albatrellus confluens* (Alb. and Schwein.) Kotl. and Pouzar and structurally confirmed, their inhibitory activity was validated *in vitro*, and their metabolic effects were tested in a mouse model of diet-induced obesity. It was found they can act as sphingomyelin synthase inhibitors, prevent weight gain, and improve vitamin D homeostasis. Resveratrol is a natural polyphenolic compound. Lv et al. presented a comprehensive and systematic review on resveratrol intervention in animal models of retinal diseases, providing preclinical support for its possible therapeutic uses in the management of retinal diseases. Formononetin is a common natural metabolite; Jin et al. presented a review on its sources, pharmacological activities and molecular mechanisms, co-administration, toxicity, derivatives, and drug delivery systems over the last 5 years. Royal jelly acid (10-HDA) is an unsaturated fatty acid unique to royal jelly—Zhi et al. contributed a review on its preparation, metabolism, and potential pharmacological activities in managing cancer, inflammatory disorders, and glucolipid metabolic diseases.

In the area of traditional Chinese medicine, Liu et al. present the development and preliminary mechanistic analysis of compound ganoderma lucidum hepatoprotective effervescent granules. Liu et al. reviewed the natural metabolites from medicinal plants used in traditional Chinese medicine for cardiovascular diseases by highlighting pharmacological mechanisms, evidence, and future directions; specifically, they suggest how traditional Chinese medicine-derived therapies could be integrated into cardiovascular care as a novel multi-target approach.

## 2 Concluding remarks

People from different cultural backgrounds and biogeographic regions have different strategies for sustaining their lives and avoiding hunger and illness. Plants have proven to be common substances for food and/or medicine. In this Research Topic, contributors report the plants used for medicinal purposes, reveal their active compounds, and indicate their potential health uses. Moreover, several species used on a global scale are also presented. This shows that the framework of cross-cultural assembly of traditional plant knowledge will benefit global health. Ethnopharmacology is still a reliable way to demystify the health-promoting mechanism of food-medicinal plants and is thus proof of the ‘food and medicine continuum’.
